# Cannabidiol Induces Cell Death in Human Lung Cancer Cells and Cancer Stem Cells

**DOI:** 10.3390/ph14111169

**Published:** 2021-11-17

**Authors:** Hussein Hamad, Birgitte Brinkmann Olsen

**Affiliations:** 1 Department of Nuclear Medicine, Odense University Hospital, 5000 Odense C, Denmark; hussein_hamad17@live.dk; 2 Department of Clinical Research, University of Southern Denmark, 5000 Odense C, Denmark

**Keywords:** lung cancer, cancer stem cells, cannabidiol, reactive oxygen species, apoptosis

## Abstract

Currently, there is no effective therapy against lung cancer due to the development of resistance. Resistance contributes to disease progression, recurrence, and mortality. The presence of so-called cancer stem cells could explain the ineffectiveness of conventional treatment, and the development of successful cancer treatment depends on the targeting also of cancer stem cells. Cannabidiol (CBD) is a cannabinoid with anti-tumor properties. However, the effects on cancer stem cells are not well understood. The effects of CBD were evaluated in spheres enriched in lung cancer stem cells and adherent lung cancer cells. We found that CBD decreased viability and induced cell death in both cell populations. Furthermore, we found that CBD activated the effector caspases 3/7, increased the expression of pro-apoptotic proteins, increased the levels of reactive oxygen species, as well as a leading to a loss of mitochondrial membrane potential in both populations. We also found that CBD decreased self-renewal, a hallmark of cancer stem cells. Overall, our results suggest that CBD is effective against the otherwise treatment-resistant cancer stem cells and joins a growing list of compounds effective against cancer stem cells. The effects and mechanisms of CBD in cancer stem cells should be further explored to find their Achilles heel.

## 1. Introduction

Lung cancer remains the leading cause of cancer-related deaths [1]. Lung cancer is divided into two main subtypes: small cell lung cancer (SCLC) and non-small cell lung cancer (NSCLC), where the latter is the most prevalent and accounts for more than 80% of lung cancers. Survival remains poor because most patients (approx. 75%) have advanced disease at diagnosis and, many patients relapse after surgery with a poor prognosis [2]. The effects of conventional anti-lung cancer treatments such as radio- and chemotherapy and targeted therapy are often limited by resistance development [3].

Subpopulations of lung cancer cells with stem-like characteristics, the so-called cancer stem cells or cancer stem-like cells, have been associated with resistance to therapy and thus relapse [4,5,6]. Targeting the cancer stem cells should be the goal for successful therapy. However, as the cancer stem cell phenotype is dynamic, it will be necessary to target and eliminate all cancer cells to achieve complete remission [7,8]. The reasons behind therapy resistance in cancer stem cells include quiescence (dormancy), increased DNA repair, increased drug efflux, high expression of anti-apoptotic proteins, and increased expression of detoxifying enzymes, i.e., free radical scavengers [9,10,11,12,13].

Cannabidiol (CBD) is an abundant cannabinoid found in the Cannabis sativa plant [14]. Cannabinoids have anti-cancer effects in vitro and in vivo in several different cancers, including glioma, breast, lung, cervical, liver, thyroid, colorectal, prostate, gastric, and leukemia/lymphoma (reviewed in [15]). CBD modulates the activity of orphan and de-orphan G-protein coupled receptors (GPCRs) such as 5HT1A and GRP55 and non-GPCRs such as TRPVs and PPARγ (reviewed in [16]).

Although it has been shown that CBD has anti-cancer effects on cancer cells, not much is known about the effect of CBD on cancer stem cells. One report in glioblastoma stem cells has shown that, in vitro, CBD increased reactive oxygen species, decreased survival, and inhibited self-renewal. In vivo, CBD improved the survival of glioblastoma xenografts [17]. Here, we show that CBD reduced viability in both lung cancer stem cells and adherent lung cancer cells. Furthermore, we show that CBD induced cell death, reduced sphere formation, and increased oxidative stress and mitochondrial membrane potential in cancer stem cells and adherent cancer cells.

## 2. Results

**Figure 1:**
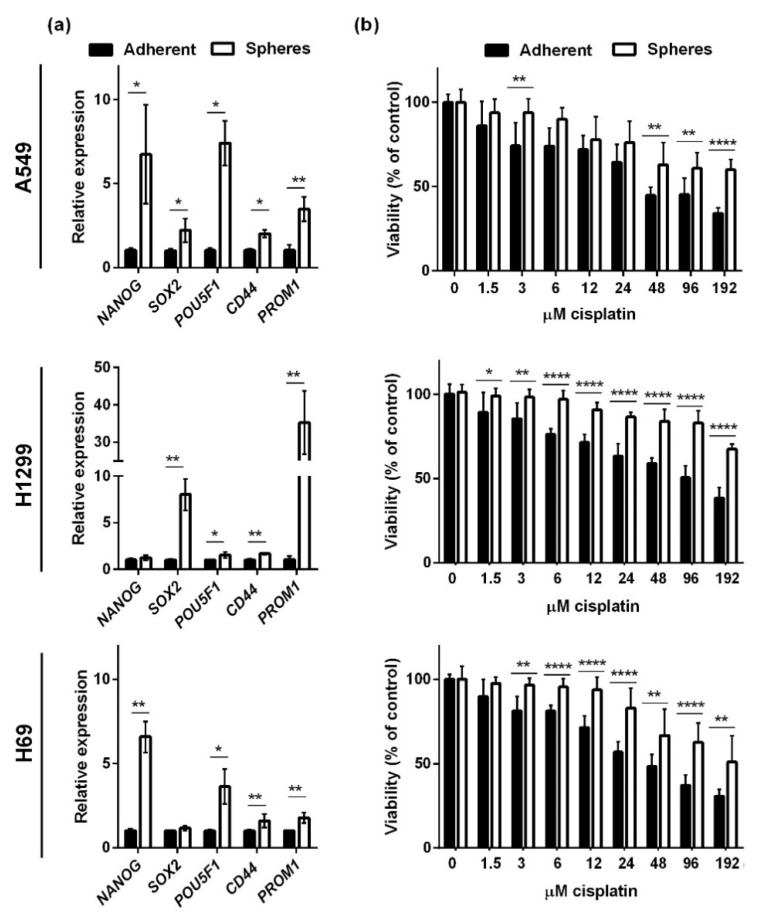
Spheroid cells express higher levels of stemness markers and are more resistant to cisplatin than adherent cells. Lung cancer cells were grown as adherent cells or spheres for 72 h. The spheroid cells expressed higher levels of the stemness markers *NANOG*, *POU5F1* (Oct4), *SOX2*, *CD44*, and *PROM1* (CD133). The results were normalized to the housekeeping genes *HRPT1* and *GAPDH* and compared to adherent cells, whose expression level was set to 1. * *p* < 0.05; ** *p* < 0.01 as determined by *t* test (**a**). Spheroids or adherent cells were incubated with increasing concentrations of cisplatin (0–192 µM) for 24 h. Cell viability was measured with CellTiter-Blue and normalized to either untreated adherent cells or spheres (**b**). Results are shown as the mean ± SD of three independent experiments. * *p* < 0.05; ** *p* < 0.01; **** *p* < 0.0001 compared to adherent cells as determined by *t* test.

We analyzed the effects of CBD in two NSCLC cell lines (A549 and H1299) and an SCLC cell line (H69). The two NSCLC cells lines grow as adherent cells in the presence of serum, whereas H69 in the presence of serum grow as floating aggregates (although we refer to them as adherent in the following). Under non-adherent, serum-free conditions, bulk cancer cells undergo cell death by anoikis, whereas the cancer stem cells survive and give rise to spheres enriched in cancer stem cells [18,19,20]. To verify that the spheres in our setup were enriched in cancer stem cells, we tested the expression of the stemness-related transcription factors *POU5F1* (Oct4), *SOX2*, *CD44*, *PROM1* (CD133), and *NANOG* in spheres and adherent cells 72 h after seeding (Figure 1a). We normalized the expression levels to the housekeeping genes *GAPDH* and *HPRT1* and found that sphere conditions significantly increased the expression of *NANOG*, *SOX2*, *POU5F1*, *CD44,* and *PROM1* in A549 cells (Figure 1a, upper panel). In H1299 spheres, there was a significantly increased expression of *SOX2*, *POU5F1*, *CD44,* and most pronounced *PROM1* (Figure 1a, middle panel). In the SCLC spheres (H69), all stemness markers except *SOX2* were significantly upregulated (Figure 1a, lower panel). Since cancer stem cells are more resistant to conventional therapy, we tested the viability of the spheres and adherent cells after 24 h incubation with cisplatin (Figure 1b). We saw a dose-dependent decrease in all three cell lines, but the spheres were more resistant to cisplatin, especially at the higher concentrations. Overall, these results suggest that the spheres were enriched in cancer stem cells.

**Figure 2:**
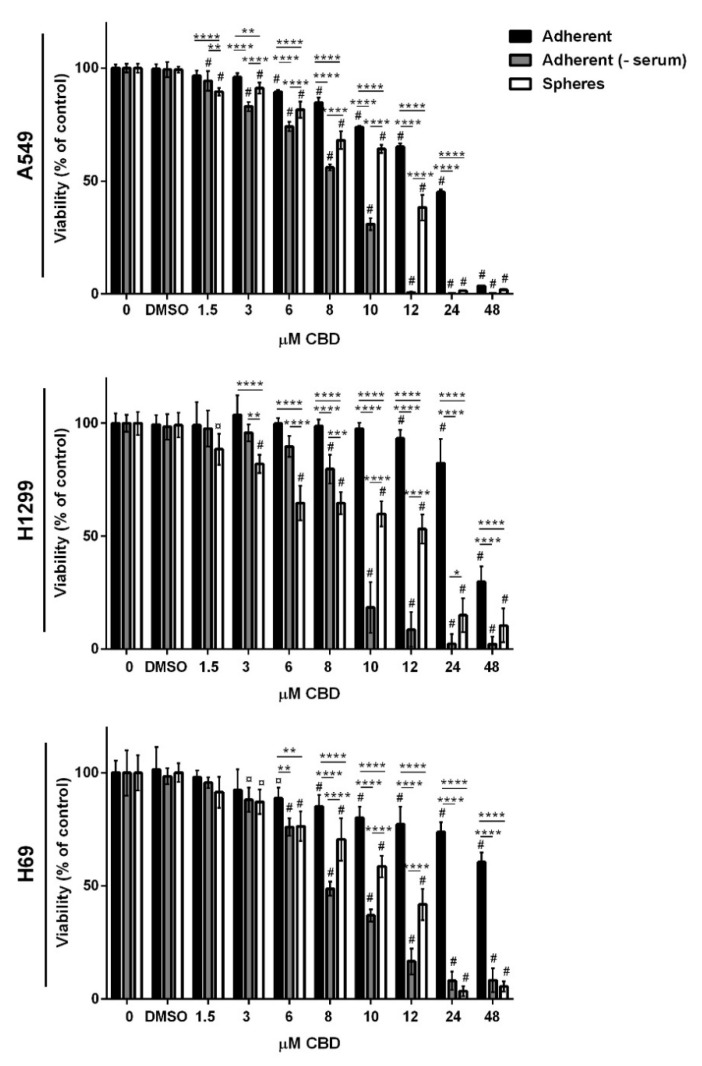
CBD decreases viability. Adherent cells in the presence or absence of serum or spheres were incubated with increasing CBD concentrations (0–48 µM) for 24 h, and the viability was determined by the CellTiter-Blue assay. The results were normalized to adherent cells with or without serum or spheres incubated without CBD and shown as the mean ± SD from triplicates of three independent experiments. Differences of means to control were significant (*p* < 0.001 or *p* < 0.05) as indicated by # or ¤, respectively. Differences of means between the populations for the individual CBD concentrations are indicated by asterisks. ** *p* < 0.01, *** *p* < 0.001, **** *p* < 0.0001 as determined by two-way ANOVA using Tukey.

Next, we tested the effect of CBD on viability in spheres and adherent cells in the presence and absence of serum (Figure 2). Cells were seeded 72 h before adding increasing concentrations (0–48 µM) of CBD. After 24 h incubation, cell viability was measured using CellTiter-Blue. CBD induced a significant dose-dependent decrease in viability in all three populations in all three cell lines.

The spheres were significantly more sensitive than when cells were grown in the presence of serum at all doses tested, except at 48 µM in A549 cells, where almost no cells were viable in either population (Figure 2). The results showed that in the absence of serum, the effects of CBD were much stronger than when serum was present (Figure 2) since at 10 µM, there were only approximately 30–40% viable cells, and at 12 µM, there were even fewer viable cells left. We also tested the effect of DMSO in which CBD was dissolved, but there was no effect on viability. Overall, the results showed that CBD reduces the viability of both adherent lung cancer cells and lung cancer stem cells and that the effect is affected by serum in the medium.

We also tested the effects of CBD on A549 cells grown in 0%, 2.5%, 5%, and 10% FBS (Supplementary Figure S1) and found that 5% were not different from 10% FBS, whereas in 2.5% of FBS, the effects of CBD were more pronounced, but CBD was not as effective as when serum was absent.

**Figure 3:**
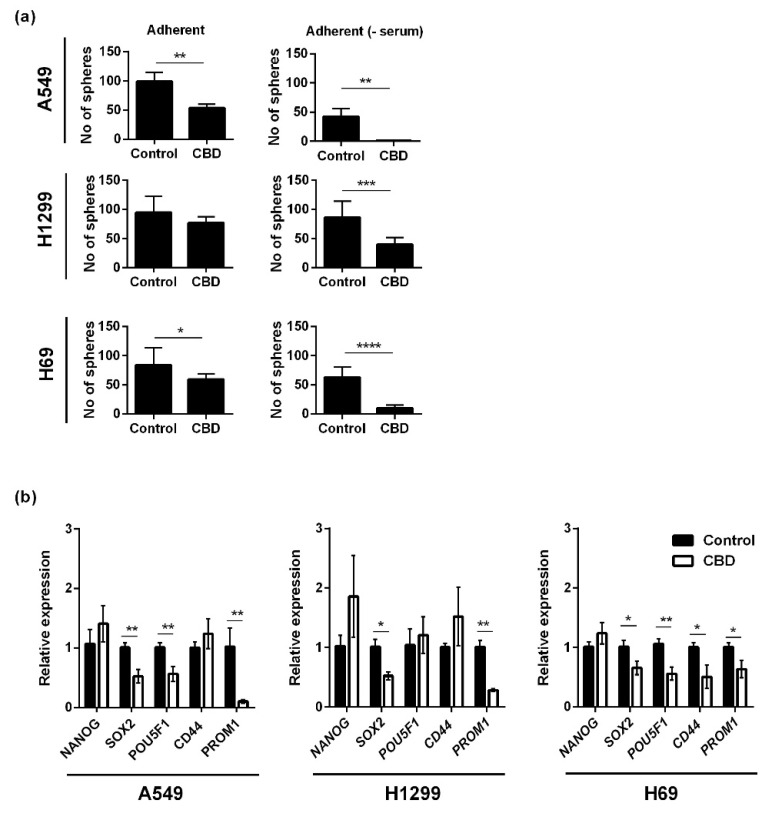
CBD decreases sphere formation and the expression of cancer stem cell genes. Adherent cells were treated with 10 µM CBD for 24 h in the presence or absence of serum. Next, the cells were trypsinized and seeded as spheres and incubated for 7 days and counted (**a**). The result is shown as the mean number of spheres ± SD of three independent experiments. * *p* < 0.05, ** *p* < 0.01, *** *p* < 0.001, **** *p* < 0.0001 as determined by *t* test. Lung cancer spheres were incubated with 10 µM CBD for 24 h, and the expression of the cancer stem cells genes *NANOG*, *SOX2*, *POU5F1* (Oct4), *CD44,* and *PROM1* (CD133) were analyzed and normalized to the housekeeping genes *HPRT1* and *GAPDH* and compared to control cells, whose expression level was set to 1 (**b**). The results are shown as the mean ± SD of three independent experiments. * *p* < 0.05, ** *p* < 0.01 as determined by *t* test.

Based on the viability results, where we saw an effect also on the spheres, we tested the effect of CBD on sphere formation. Adherent lung cancer cells were incubated with 10 μM CBD for 24 h in the presence or absence of serum, and afterward, the cells were seeded in non-adherent plates in serum-free medium for seven days and counted. For A549, the number of spheres was significantly reduced after treatment with CBD to approximately 50% of the control (*p* < 0.01) when the incubation took place in serum-containing medium and by approximately 10-fold in the absence of serum (*p* < 0.01; Figure 3a, upper panel). For the H1299 cells, sphere formation was also significantly reduced by CBD (*p* < 0.001) but only in the absence of serum (Figure 3a, middle panel). In H69 cells, sphere formation was reduced in the presence of serum (*p* < 0.05) and even more so in the absence of serum (*p* < 0.0001; Figure 3a, lower panel). Overall, the results suggest that CBD inhibits sphere formation, i.e., self-renewal. We also tested the effect of CBD on the expression level of five stemness-related genes (*NANOG*, *SOX2*, *POU5F1, CD44,* and *PROM1*) in the spheres using qPCR. In A549 spheres, the expression levels of *SOX2*, *POU5F1*, and *PROM1* decreased significantly, in particular *PROM1* (*p* < 0.01), whereas *NANOG* and *CD44* increased, although not significant (Figure 3b, left panel) In H1299 spheres, CBD reduced the expression of *SOX2* (*p* < 0.05) and *PROM1* (*p* < 0.01; Figure 3b, middle panel). In H69 spheres, CBD significantly reduced the expression of *SOX2*, *CD44* and *PROM1* (*p* < 0.05) and *POU5F1* (*p* < 0.01; Figure 3b, right panel).

**Figure 4:**
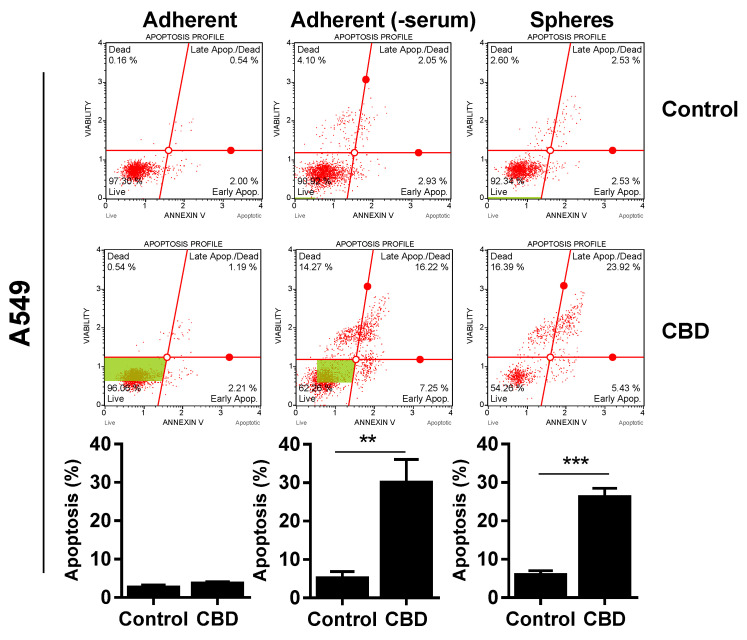

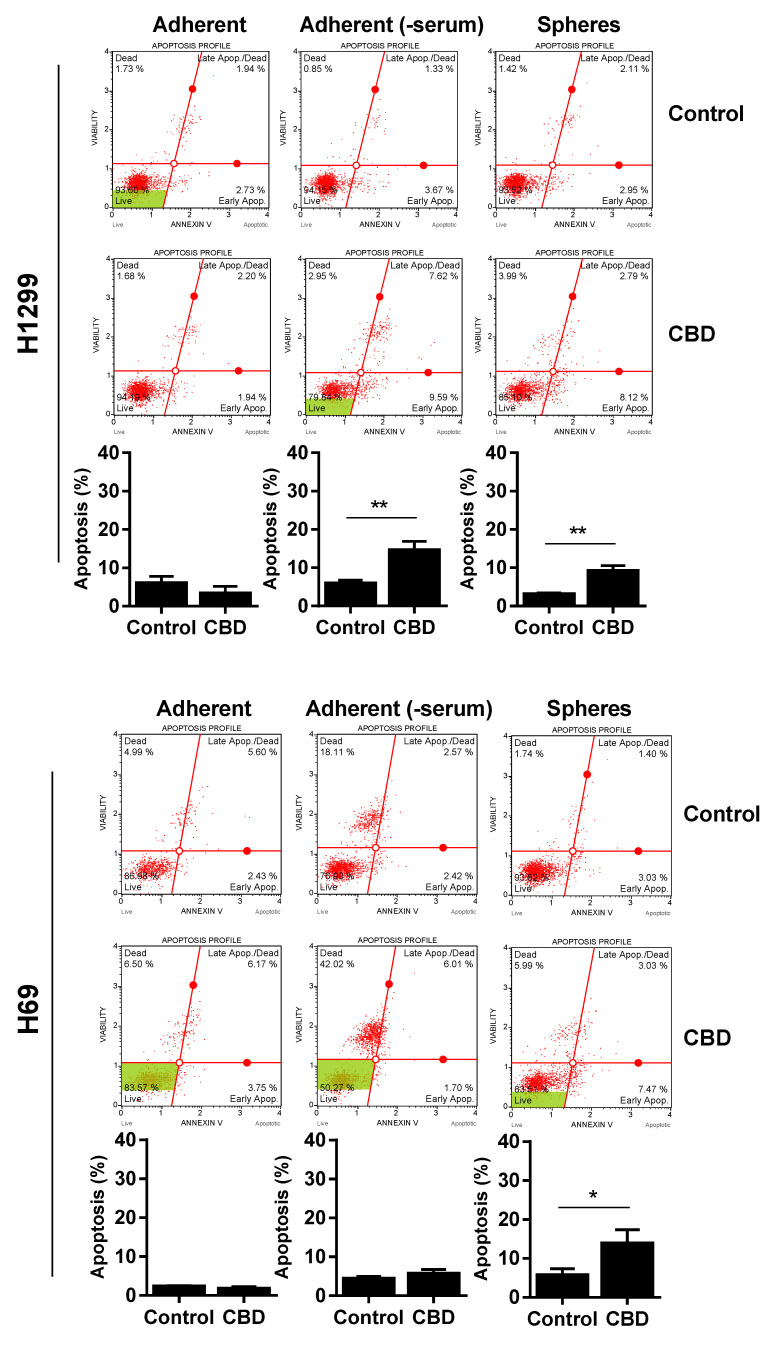
CBD induces cell death. Adherent cells in the presence and absence of serum or spheres were incubated with 10 µM CBD for 24 h. Cells were stained with Annexin V/7-AAD and analyzed on the MUSE Cell Analyzer. The Annexin-positive cells represent apoptotic cells (early + late apoptosis). Results are shown as mean ± SD of three independent experiments. * *p* < 0.05; ** *p* < 0.01; *** *p* < 0.001 compared to control cells as determined by *t* test.

Since CBD reduced viability, we wanted to determine if the effect was cytotoxic, so we performed an AnnexinV and cell death analysis. For A549, Annexin-positive cells significantly increased to approx. 30% after 24 h of 10 µM in the spheres (Figure 4, upper panel) and adherent cells incubated in the absence of serum (Figure 4, upper panel). We did not see any significant apoptosis in the adherent cells in the presence of serum (Figure 4, upper panel).

There was also a significant increase in apoptotic cells in the H1299 spheres and H1299 cells grown without serum, although the increase was smaller (increased to approx. 10–15%; *p* < 0.01; Figure 4, middle panel). For H69, there was only a significant increase in apoptotic cells in the spheres (*p* < 0.05), whereas cells without serum were not Annexin-positive (Figure 4, lower panel). In contrast, there was a pronounced increase in dead cells negative for Annexin, especially in the H69 cells grown without serum. Overall, these results suggest that CBD induces apoptosis in the lung cancer spheres.

**Figure 5:**
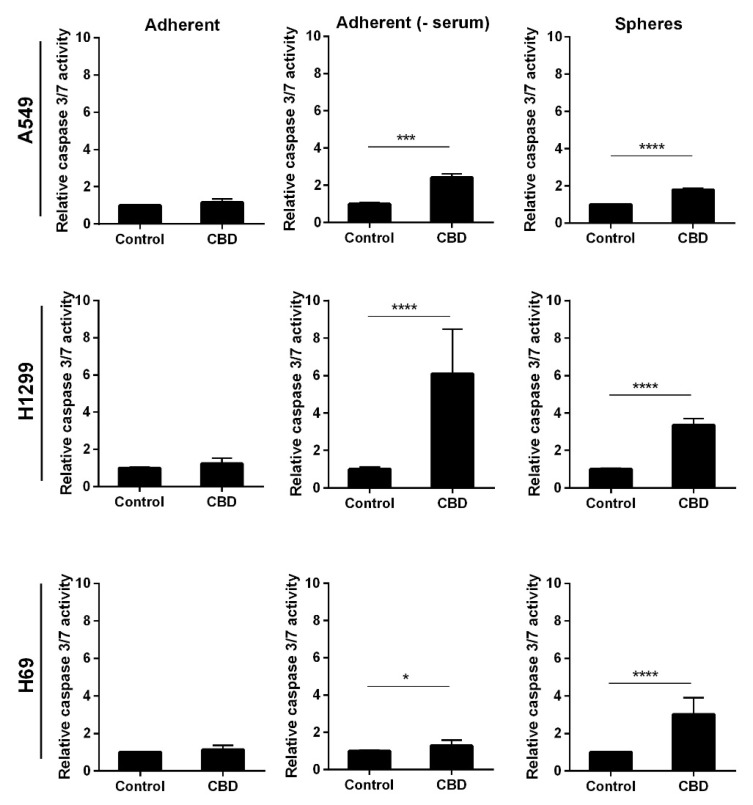
CBD activates caspase 3/7. Adherent cells in the presence and absence of serum or as spheres were incubated with 10 µM CBD for 24 h, and the activity of caspase 3/7 was measured by luminescence. The results were normalized to control cells and are shown as the mean ± SD of three independent experiments. * *p* < 0.05, *** *p* < 0.001; **** *p* < 0.0001 compared to control cells as determined by *t* test.

Next, we analyzed whether the apoptosis was caspase-mediated by incubating adherent and non-adherent cells with 10 µM CBD for 24 h. In the absence of serum, the adherent and the non-adherent A549 cells showed a two-fold increase in caspase 3/7 activity (*p* < 0.001 and *p* < 0.0001, respectively (Figure 5, upper panel)). Whereas in the adherent cells in the presence of serum, we did not observe an increase (Figure 5, upper panel). In H1299 and H69, CBD led to significant activation of caspase 3/7 in the spheres as well as the adherent H1299 cells without serum (*p* < 0.0001) and a much smaller, though significant, increase in the H69 cells in the absence of serum, whereas there was no caspase activation in the presence of serum (Figure 5, middle and lower panel). These results indicate that CBD activates caspase-dependent apoptosis in the spheres and the adherent cells in the absence of serum.

**Figure 6:**
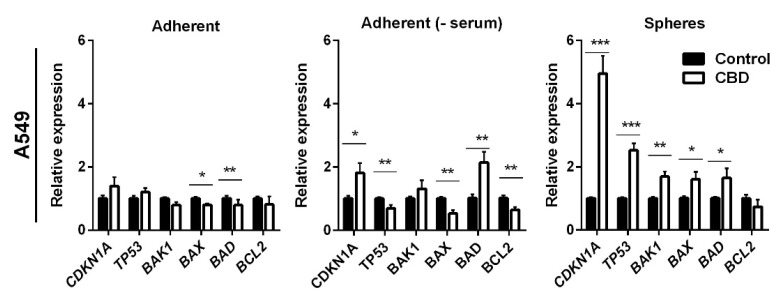
CBD induces changes in the expression of apoptotic genes. Adherent cells in the presence and absence of serum or as spheres were incubated with 10 µM CBD for 24 h. The expression levels of selected anti- and pro-apoptotic genes (*BAD*, *BCL2*, *BAX*, *BAK1*), as well as *TP53* (p53) and *CDKN1A* (p21), were analyzed and normalized to the housekeeping genes *HPRT1* and *GAPDH* and compared to control cells, whose expression level was set to 1. The results are shown as the mean ± SD (n = 3). * *p* < 0.05; ** *p* < 0.01; *** *p* < 0.001 as determined by *t* test.

To further delineate the mechanism by which CBD induced apoptosis, we analyzed the expression of selected anti- and pro-apoptotic genes in A549 cells after treatment with 10 μM CBD for 24 h. We also included *TP53* and its target gene p21 (*CDNK1A*), which previously were shown to be affected by CBD [21,22]. In the adherent cells in the presence of serum, CBD decreased *BAK1* and *BAX* expression by 1.25-fold (*p* < 0.05 for *BAK1* and *p* < 0.01 for *BAX1*; Figure 6, left panel), whereas there were no significant changes in the expression of *BCL2*, *BAD*, *CDKN1A*, and *TP53*. In the adherent cells, in the absence of serum, CBD increased the expression levels of *CDKN1A* and *BAD* two-fold (Figure 6, middle panel). Furthermore, the expression of *TP53*, *BAX*, and *BCL2* decreased significantly (*p* < 0.01) in the adherent cells in the absence of serum. In the non-adherent cells, the pro-apoptotic genes *BAK1*, *BAX*, and *BAD* were significantly upregulated by approximately 1.8-fold (*p* < 0.01 for *BAK1* and *p* < 0.05 for *BAX* and *BAD*). In contrast, the expression of the anti-apoptotic *BCL2* slightly decreased, although not significantly (Figure 6, right panel). CBD induced a three-fold increase in the expression of *TP53* (*p* < 0.001) and a five-fold increase in its target gene *CDKN1A* (*p* < 0.001) in the non-adherent cells (Figure 6, left panel). *TP53* is upregulated, among other things, in response to DNA damage; however, we could not detect any phospho-H2AX foci (a marker for double-strand DNA breaks) in A549 cells at 24 h (Supplementary Figure S2). Although, we also detected a significant increase in the cyclin-dependent kinase inhibitor *CDKN1A* expression level, we could not detect a change in cell cycle distribution (results not shown).

**Figure 7:**
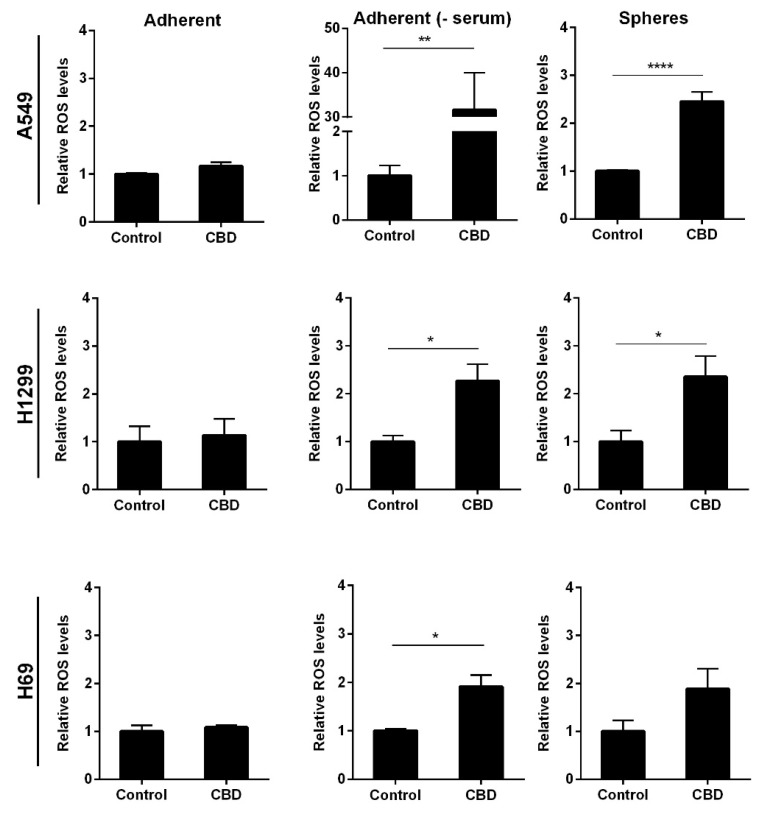
CBD increases the levels of ROS. Adherent cells in the presence, absence, or spheres were incubated with 10 µM CBD for 24 h. The levels of superoxide radicals were evaluated using luminescence and presented as relative ROS levels compared to control cells ± SD of three independent experiments. * *p* < 0.05, ** *p* < 0.01; **** *p* < 0.0001 compared to control cells as determined by *t* test.

It has previously been shown that CBD can induce oxidative stress in adherent cancer cells [22,23] and glioblastoma stem cells [17]. To investigate whether this was also the case in the lung cancer stem cells, we analyzed reactive oxygen species (ROS) levels after 24 h incubation with 10 μM CBD. We observed a significant 2.5-fold increase in ROS levels in the non-adherent A549 and H1299 cells (*p* < 0.0001 and *p* < 0.05) compared to control cells (Figure 7 upper and middle panel). These results indicate that 10 µM CBD induces oxidative stress in the non-adherent NSCLC cells. In the non-adherent H69 cells, the increase was not significant (Figure 7, lower panel). There was no measurable oxidative stress in the adherent cells in the presence of serum in either of the tested cell lines (Figure 7, left panel). In the absence of serum, we measured a 30-fold increase in ROS levels in A549 cells (*p* < 0.01) but only approx. 2.5- and 2-fold in H1299 and H69 cells, respectively (*p* < 0.05; Figure 7, middle panel), suggesting that oxidative stress is massively induced in adherent A549 cells when serum is not present but more moderately induced in the other two cell lines.

**Figure 8:**
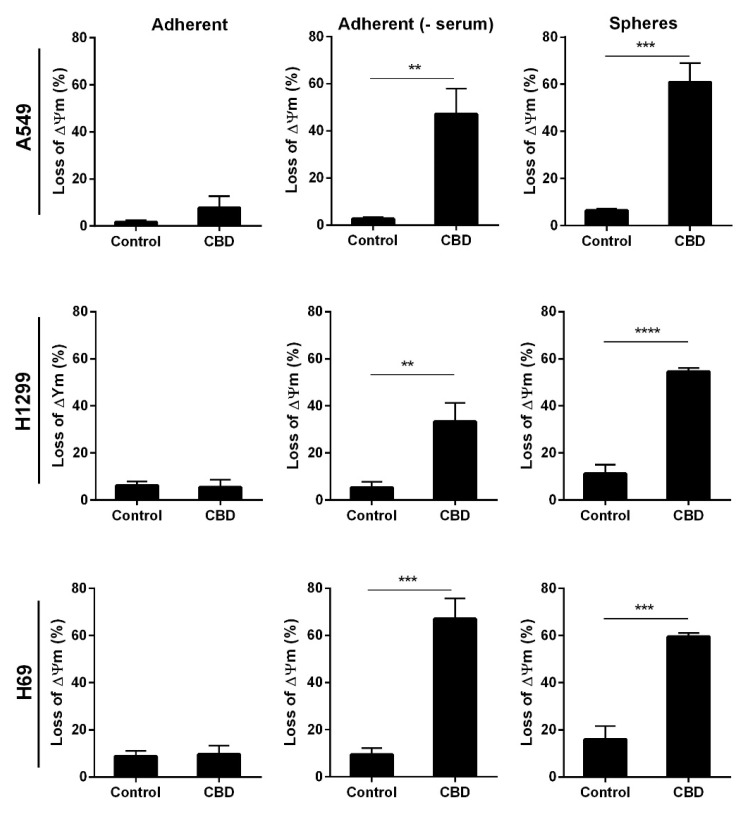
CBD decreases the mitochondrial membrane potential. Adherent cells in the presence, absence, or spheres were incubated with 10 µM CBD for 24 h. Next, cells were stained with JC-1 to measure the mitochondrial membrane potential. Data were analyzed using the NucleoCounter NC-3000. The result is the mean of cells with low mitochondrial membrane potential ± SD of three independent experiments. ** *p* < 0.01, *** *p* < 0.001 **** *p* < 0.0001 compared to control cells as determined by *t* test.

Finally, we tested the mitochondrial membrane potential since the destruction of mitochondria can lead to increased oxidative stress. Loss of mitochondrial membrane indicates healthy intact mitochondria, and low membrane potential indicates permeable mitochondria. Adherent and non-adherent cells were treated with 10 μM CBD for 24 h and analyzed. There was a more significant loss in mitochondrial membrane potential (60%) in all the non-adherent cells compared to control cells (*p* < 0.001 and *p* < 0.0001, Figure 8, right panel), whereas the adherent cells in the presence of serum remained unaffected (Figure 8, left panel). For the adherent A549 cells, in the absence of serum, there was a 45% loss of membrane potential, a 40% loss in H1299 cells, and a 70% loss in H69 cells (Figure 8, middle panel). These results support that CBD leads to mitochondrial damage in the adherent cells in the absence of serum and in the spheres.

## 3. Discussion

Lung cancer patients experience resistance to treatment and recurrence attributed to cancer stem cells [4,5,6]. Therefore, it is essential to find new treatment strategies that also effectively target this subpopulation as current therapy cannot. The effects of CBD on cancer cell lines have been extensively investigated, but not much is known of the effects on resistant cancer stem cells. Here, we sought to analyze the effect of CBD on resistant lung cancer spheres enriched in cancer stem cells compared to adherent lung cancer cells. We found that CBD reduced viability and induced cell death and increased oxidative stress, and led to a loss of mitochondrial membrane potential in cancer stem cell-enriched spheres and the adherent cells in the absence of serum.

Non-adherent three-dimensional cell culture in a serum-free defined medium is a common technique based on self-renewal and anoikis resistance. It is based on neurospheres but has since been used in several cancer types, including lung cancer [24,25,26]. The technique enriches for cells with stemness features, including self-renewal, unlimited growth abilities, tumorigenic potential in vivo, ability to differentiate, high invasion capacity, and resistance to high doses of chemotherapy [25,26]. Furthermore, the technique is marker-independent and eliminates the use for unique markers whose identification in lung cancer stem cells remains challenging [27,28,29].

The spheres were grown in serum-free medium and, when the adherent cells were tested in serum-free medium, we observed a reduction in viability at lower CBD concentrations than cells grown in the presence of serum. Interestingly, CBD had the same effect on viability at 5% and 10% serum, whereas at 2.5% the effect of CBD was more pronounced but still not as effective as the absence of serum. This agrees with earlier reports of lower CBD efficacy in serum [30], probably due to the binding of CBD to albumin [31].

CBD inhibited self-renewal, consistent with results from glioblastoma [17]. The effect on sphere formation was more pronounced than the effect in the viability experiments. Whereas the viability assay measures the metabolic activity, the sphere formation assay analyzes self-renewal, a hallmark of only the cancer stem cells [32]. Mechanistically, we found that CBD reduced the expression of stemness-related transcription factors *SOX2* and *POU5F1* in the non-adherent A549 and H69 cells, consistent with reduced sphere formation ability, whereas *NANOG* increased, and also *CD44* in H1299, suggesting differences in the cell lines. It was previously shown in breast cancer stem cells that decreased G-protein-coupled receptor 3, of which CBD is an inverse agonist, increased the expression of *NANOG* [33], which could explain the increase in *NANOG* in response to CBD. H1299 only showed a decrease in *SOX2* and *PROM1*, and interestingly these genes showed the highest upregulation in the spheres. CBD induced a significant increase in *TP53* and *CDKN1A* in cancer stem cells, whereas the increase in *CDKN1A* was much smaller in the adherent cells, and *TP53* expression even decreased in the adherent cells. These results suggest different mechanisms in the two populations. We speculate that the difference concerning p53 expression is due to the serum status of the medium. Although the sphere medium lacks serum, it does contain growth factors. The serum status could also explain the differences found by others, as some publications report decreased p53 expression in response to CBD [22,34], whereas Lukhele et al. found that CBD increased p53 expression [21].

Several studies have shown that CBD induces apoptosis in cancer cell lines [17,29,35,36,37]. Here, we show that CBD also induced apoptosis in the lung cancer spheres, enriched in cancer stem cells. CBD induced the highest percentage of apoptosis in A549 cells and the least in H1299 cells. Interestingly, CBD did not induce apoptosis in H69 cells grown in the absence of serum, although we did observe apoptosis in the H69 spheres. CBD also led to a significant increase in the expression of pro-apoptotic *BAK1*, *BAX*, and *BAD* in the non-adherent A549 cells, supporting a role for intrinsic mitochondrial apoptosis in cancer stem cells in response to CBD, as reported by others in cancer cells [21,23]. Bcl2 forms a complex with Bad, leading to inhibition of Bcl2, which causes Bax and Bak1 to translocate to the mitochondria and contribute to apoptosis; however, we did not see a significant decrease in *BCL2* expression in the A549 cancer stem cells. There was a significant increase in *BAD* and a significant decrease in *BAX* and *BCL2* expressions in the adherent A549 cells. The difference could be explained by the aberrant expression levels of pro- and anti-apoptotic proteins in adherent and non-adherent cells [38].

Further supporting the induction of apoptosis was the activation of effector caspases 3/7. Previous studies have shown that CBD increased caspase activity in cancer cells [29,37,39,40,41]. Singer et al. also showed that CBD activated caspase 3 in nude mice implanted with glioblastoma stem cells [17]. Overall, our results support that CBD induces caspase-mediated cell death also in cancer stem cells, but consistent with the Annexin results, there was only a very modest activation of caspase 3/7 in H69 cells in the absence of serum, supporting the notion that the effects of CBD are cell-dependent

CBD induced massive oxidative stress in the adherent A549 cells in agreement with the literature [22,23,37]. However, there was also induction of oxidative stress in the A549 spheres, albeit not as massive. The observation that CBD caused less oxidative stress in the spheres was only seen for the A549 cells. CBD induced similar oxidative stress in adherent and non-adherent H1299 and H69 cell lines, supporting cell-context specific effects. In H69 cells, the absence of serum itself caused an increase in ROS levels, whereas ROS was not affected by serum in A549 and H1299 cells. Cancer stem cells have been shown to harbor lower ROS levels than non-stem cancer cells due to lower ROS production or a higher expression of anti-oxidant genes [11]. A higher expression of anti-oxidant genes could explain the smaller increase in ROS levels in response to CBD in spheroid A549 cells but warrants further investigations. Interestingly. CBD exhibits both pro-and anti-oxidant functions (reviewed in [42]) dependent on CBD dose, incubation time, and more importantly, cell type. With respect to the H69 and H1299 cells, there was a modest increase in ROS levels in response to CBD both in adherent cells and in the spheres, comparable to the A549 spheres, again supporting that the effects of CBD are cell context-dependent.

We did not detect any DNA double-strand breaks induced by CBD. However, it has been reported that CBD could induce DNA damage (strand breaks and oxidative damage) in buccal and liver cancer cells [43], whereas others did not detect any DNA damage in colon cancer cells [44]. Phospho-H2AX is an early marker for DNA double-strand breaks, and it would be interesting to analyze them at an earlier time-point than 24 h. Furthermore, Russo et al. [43] used a comet assay, which does not distinguish between single- and double-strand breaks, whereas phospho-H2AX is specific for double-strand breaks, so we cannot exclude that CBD induces other forms of DNA damage in the lung cancer cells.

CBD affects many cellular targets, and although there is no consensus on its precise mode of action, CBD appears to influence mitochondrial function. We found that CBD induced considerable loss of mitochondrial membrane potential in both populations in all three cell lines, albeit most pronounced in cancer stem cells, with the exception of H69. It was previously shown that CBD decreased membrane potential [22] in cancer cell lines, and CBD directly targeted the mitochondria in leukemia cells [37]. More specifically, it has been shown that CBD interacted with and modulated the voltage-dependent anion channel 1 (VDAC1) located in the mitochondrial outer membrane [45]. In leukemic cells, CBD and VDAC interaction led to increased Ca2+ influx into the mitochondria, ultimately leading to cytochrome c release and apoptosis [37]. We found that CBD reduced mitochondrial potential, and we also observed increased oxidative stress, caspase activation, and apoptosis, reactions where mitochondria play a role. Whether the mitochondrial dysfunction is the cause or the consequence remains to be established by a direct assay of mitochondrial function.

When H69 cells in the absence of serum were incubated with CBD, we did not detect an increase in apoptosis or caspase-activation but a large increase in ROS. From the cell death analyses, we observed an increase in dead H69 cells not positive for Annexin. It was previously shown that CBD induced autophagy-dependent cell death [23]. However, other cell death pathways cannot be excluded, e.g., pyroptosis, as shown in primary liver cancer cells in response to CBD [46]. CBD also induced MPT-driven necrosis (mitochondrial permeability transition) in leukemic cells, initiated by severe oxidative stress and Ca^2+^ overload [37].

Cancer stem cells have increased mitochondrial mass and overall increased mitochondrial function [47]. Reduction of mitochondrial membrane potential, overproduction of ROS, and inhibition of mitochondrial biogenesis affect the survival of cancer stem cells [48]. This makes mitochondria an attractive target for cancer stem cells eradication; however, only a few compounds have been identified targeting cancer stem cells [49,50,51]. Examples include salinomycin, metformin, and parthenolide which all target mitochondria [49,50,51].

CBD not only has shown promising anti-cancer effects but also has anti-inflammatory effects [52] and effects in multiple sclerosis, Alzheimer’s, Parkinson’s, epilepsy, and chronic pain management [53]. However, the exact mechanism of action remains to be established to understand the therapeutic potential of CBD.

## 4. Materials and Methods

### 4.1. Materials

CBD (Little Green Pharma, Odense, Denmark) was dissolved in DMSO and frozen in aliquots. Just before use, an aliquot was thawed, and the CBD was diluted in prewarmed serum-free DMEM-F12. Cisplatin (Sigma) was dissolved in sterile H_2_O and frozen in aliquots.

### 4.2. Cell Culture

The human NSCLC cell lines A549 and H1299 were obtained from the American Type Culture Collection (Middlesex, UK). The SCLC line H69 was obtained from CLS Cell Lines Service (Eppelheim, Germany). The cells were grown as adherent cells in Dulbecco’s Modified Eagle’s Medium/Nutrients mixture F-12 with Glutamax (DMEM-F12) supplemented with 10% fetal bovine serum (FBS) and 1% penicillin/streptomycin (all from ThermoFisher Scientific, Roskilde, Denmark). Spheres were grown in serum-free DMEM-F12 supplemented with 1% penicillin/streptomycin, 1% Vitamin B27, 20 ng/mL epidermal growth factor (all from ThermoFisher Scientific), and 20 ng/mL basic fibroblast growth factor (Peprotech, Stockholm, Sweden). Spheres were grown in plates coated with poly(2-hydroxyethyl methacrylate) (polyhema, Sigma-Aldrich, Copenhagen, Denmark) to prevent adhesion. The cells were maintained at 37 °C under 5% CO_2_. All experiments were performed on adherent cells grown in serum-containing medium and spheres grown in serum-free medium. The serum-free adherent cells were seeded in serum-containing medium; just before the addition of CBD, the medium was changed to serum-free medium.

### 4.3. Cell Viability

Single cells were seeded in 96-well plates in a volume of 50 μL at a density of 1000 cells. After 72 h, 50 μL of increasing CBD concentrations (0–48 μM) were added as indicated in the figures. The cells were incubated for 24 h, and cell viability was evaluated by the addition of 13 μL CellTiter-Blue (Promega, Nacka, Sweden). Fluorescence was measured at (Ex_520nm_/Em_580–640nm_) in a GloMax Explorer (Promega, Nacka, Sweden). The viability was normalized to control cells and presented as percentages.

### 4.4. Sphere Formation Assay

Adherent cells were incubated with 10 μM CBD for 24 h. After treatment, the cells were trypsinized with TrypLExpress (ThermoFisher Scientific, Roskilde, Denmark), and 500 cells were seeded per well in polyhema-coated 6-well plates in serum-free medium. After 7 days, the number of spheres per well was counted under a microscope (DM 2000 LED Microscope, Leica Microsystems, Copenhagen, Denmark).

### 4.5. Cell Death and Apoptosis

After incubation with CBD for 24 h, the cells were harvested with TrypLExpress, resuspended in 1% bovine serum albumin/phosphate-buffered saline (BSA/PBS), and counted. Next, 25,000 cells were mixed with 50 μL of MUSE Annexin V & Dead Cell Reagent (Luminex, ′s-Hertogenbosch, The Netherlands) and incubated for 20 min in the dark at room temperature. The samples were diluted with 100 μL of 1% BSA/PBS and analyzed on the Guava MUSE Cell Analyzer (Luminex).

### 4.6. Caspase-Glo 3/7 Activity

Caspase activity was detected by using the Caspase-Glo 3/7 assay kit (Promega). Briefly, cells were seeded in 96-well white plates at a density of 1000 cells/well. After 72 h, 10 μM CBD was added and incubated for 24 h. A total of 100 μL of Caspase-Glo 3/7 substrate was added to the cells for 1 h before the luminescence was measured using the GloMax Explorer.

### 4.7. Reactive Oxygen Species

Cells were seeded in 96-well white plates, and after 72 h, CBD was added to a final concentration of 10 μM. After 18 h, 20 μL of H_2_O_2_ substrate solution (Promega) was added according to the manufacturer’s instructions. Cells were returned to the incubator for a further 6 h, and then 100 μL ROS-Glo Detection Solution (Promega) was added to each well. The plate was incubated for 20 min at room temperature before measuring the luminescence using the GloMax Explorer.

### 4.8. Quantitative PCR (qPCR)

Total RNA was purified using the RNeasy Plus Mini Kit (Qiagen, Copenhagen, Denmark). The RNA concentration was measured by the Qubit RNA HS kit and the Qubit 4 Fluorometer (ThermoFisher Scientific) and stored at −80 °C until use. Two μg total RNA was reverse transcribed using the RevertAid Minus First strand cDNA synthesis kit (ThermoFisher Scientific) using oligo(dT) primers, following the manufacturer’s instructions. qPCR was performed with TaqMan Fast Advanced Master Mix TaqMan assays for SOX2 (Hs01053049_s1), NANOG (Hs04260366_g1), POU5F1 (Hs0099632_g1), CD44 (Hs01075864_m1), PROM1 (Hs01009250_m1), BAD (Hs00188930_m1), BAK1 (Hs00832876_g1), BAX (Hs00180269_m1), BCL2 (Hs00608023_m1), TP53 (Hs01034249_m1), CDKN1A (Hs00355782_m1), HPRT1 (Hs02800695_m1), and GAPDH (Hs99999905_m1), all from ThermoFisher Scientific. Ten ng cDNA was used per reaction, as recommended by the manufacturer’s protocols. The qPCR was performed in a QuantStudio 3 (ThermoFisher Scientific) for 2 min at 50 °C, 2 min at 95 °C, followed by 40 cycles at 95 °C for 1 sec and 60 °C for 20 s. Expression levels were normalized to HPRT1 and GAPDH, and relative expression levels were calculated using the Pfaffl-method [54].

### 4.9. Mitochondrial Membrane Potential

Following 24 h treatment with 10 μM CBD, cells were harvested, and 50,000 cells were incubated with 3.8 μM JC-1 (5′,6,6′-tetrachloro-1,1′,3,3′-tetraethylbenzimidazolylcarbocyanine iodide, Chemometec, Allerod, Denmark) and incubated for 15 min at 37 °C. The cells were washed three times in PBS and resuspended in 15 μL 1 μg/mL DAPI (Chemometec) and loaded into an A8 cassette (Chemometec) before analysis on the NucleoCounter NC-3000 (Chemometec).

### 4.10. Statistical Analysis

Experiments were performed in triplicate. Data are shown as mean ± standard deviation (SD). Statistical significance was analyzed with *t*-test or two-way ANOVA in GraphPadPrism (GraphPad Software, San Diego, CA, USA). *p* < 0.05 was considered as a threshold of statistical significance.

## 5. Conclusions

In conclusion, we have shown that CBD is effective against treatment-resistant lung cancer stem cells, joining a growing list of compounds also effective against cancer stem cells. However, the exact mechanisms of CBD in cancer stem cells remain to be elucidated and seem to be cell context-dependent.

## Data Availability

Data is contained within the article.

## Supplementary Materials

The following are available online at https://www.mdpi.com/article/10.3390/ph14111169/s1, Figure S1: The effects of CBD are dependent on serum concentration. Figure S2: CBD does not induce phosphorylation of histone H2AX (P-H2AX). Supplementary materials and methods.
