# Supplementary material for: Cannabidiol Induces Cell Death in Human Lung Cancer Cells and Cancer Stem Cells
Source: Pharmaceuticals (Basel). 2021 Nov 17;14(11):1169. doi: 10.3390/ph14111169 (PMC8624994; doi:10.3390/ph14111169)
Supplement: Supplementary file 1 [file pharmaceuticals-14-01169-s001.zip › pharmaceuticals-1398938-supplementary.pdf]

Supplementary materials and methods

Immunofluorescence

A549 cells were incubated with 10 µM CBD in the presence of serum, absence of serum or as spheres. As a positive control, adherent cells were also incubated with cisplatin in the presence of serum. Subsequently, the cells were fixed with 4% paraformaldehyde and permeabilized with 0.2% Triton X-100. DNA double-strand breaks were visualized by incubating the cells with rabbit polyclonal anti-phospho-H2AX (P-Ser139) antibody (Sigma-Aldrich) and then with Alexafluor488-conjugated goat-anti-rabbit antibody (ThermoFisher Scientific). The cells were counterstained with DAPI (Sigma-Aldrich) and analyzed with a DM 2000 LED Microscope (Leica Microsystems).

Supplementary Figures

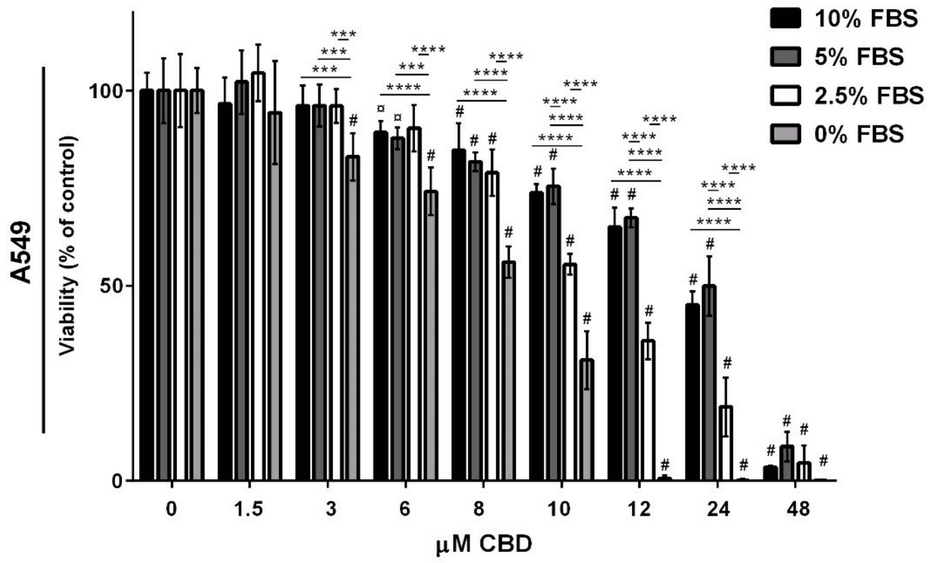

Supplementary Figure S1. The effects of CBD are dependent on serum concentration. Adherent A549 cells were incubated with increasing CBD concentrations (0-48 μM) for 24 h in 0%, 2.5%, 5% or 10% serum, and the viability was determined by the CellTiter-Blue assay. The results were normalized to control adherent cells incubated without CBD and shown as the mean +/- SD from triplicates of three independent experiments. Differences of means to control were significant ($p < 0.001$ or $p < 0.05$) as indicated by # or ¤, respectively. Differences of means between the populations for the individual CBD concentrations are indicated as by asterisks. *** $p < 0.001$, ****$p < 0.0001$ as determined by two-way ANOVA using Tukey.

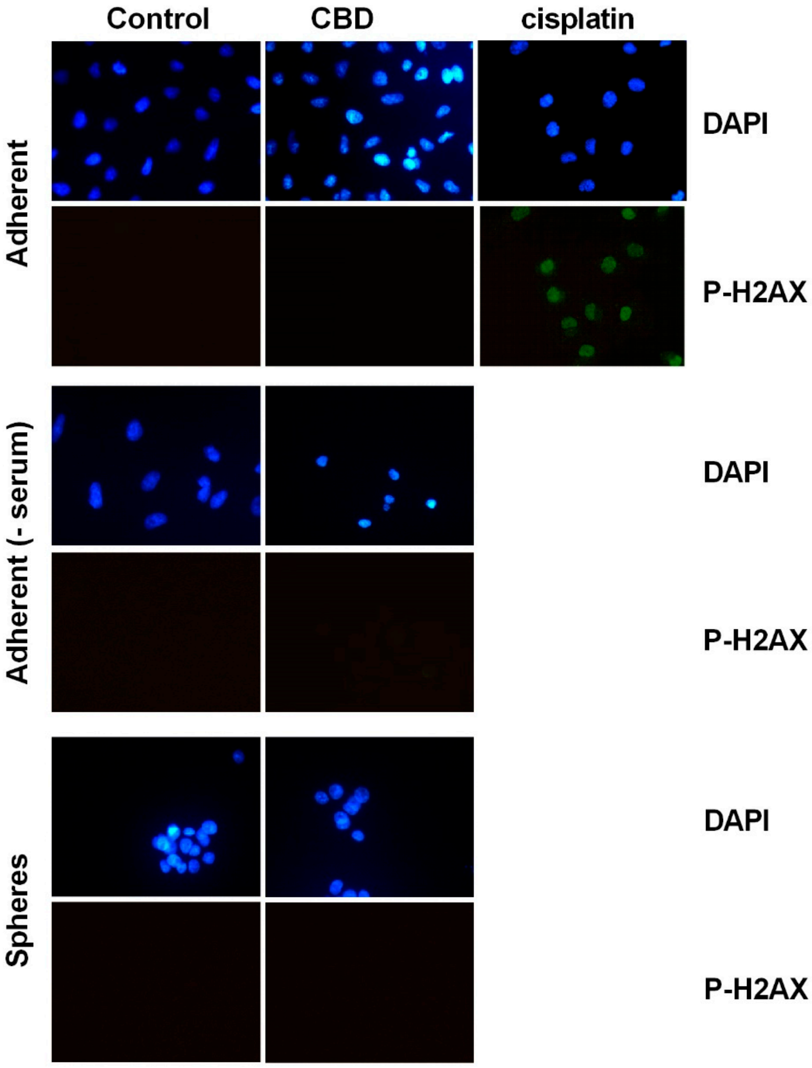

Supplementary Figure S2. CBD does not induce phosphorylation of histone H2AX (P-H2AX). Adherent A549 cells were incubated with 10 µM CBD for 24 h in the presence and absence of serum or as spheres (as a positive control cells were incubated with 10 µM cisplatin). Afterwards, cells were labeled with anti-phospho-histone H2AX antibody and with an Alexafluor488-conjugated secondary antibody (green). Cellular DNA was counterstained with DAPI (blue) and analyzed by fluorescence microscopy.
